# Supplementary material for: Digital access, transportation, and women’s empowerment in breast cancer screening uptake among Cambodian women: Analysis of the Cambodia demographic and health survey 2021–2022
Source: PLOS Digit Health. 2025 Sep 17;4(9):e0000976. doi: 10.1371/journal.pdig.0000976 (PMC12443266; doi:10.1371/journal.pdig.0000976)
Supplement: S1 Table — (DOCX) [file pdig.0000976.s001.docx]

**S1 Table**. Adjusted Odds Ratios (AOR) for Breast Cancer Screening by Smartphone Ownership and Age Group (Interaction Model)

| **Phone Ownership × Age Group** | **AOR** | **95% CI** | **p-value** |
| --- | --- | --- | --- |
| No phone × 15–29 years | Ref. | Ref. |  |
| Non-smartphone × 15–29 years | **2.06** | **1.09 – 3.89** | **0.026** |
| Smartphone × 15–29 years | 0.89 | 0.54 – 1.46 | 0.65 |
| No phone × 30–39 years | 1.26 | 0.70 – 2.25 | 0.443 |
| Non-smartphone × 30–39 years | 1.28 | 0.72 – 2.29 | 0.397 |
| Smartphone × 30–39 years | 1.71 | 1.07 – 2.75 | 0.026 |
| No phone × 40–49 years | 1.33 | 0.77 – 2.31 | 0.301 |
| Non-smartphone × 40–49 years | 1.3 | 0.75 – 2.24 | 0.35 |
| Smartphone × 40–49 years | **2.22** | **1.34 – 3.68** | **0.002** |

**Noted:** Model adjusted for media exposure, internet use, motorcycle ownership, time to care, decision-making autonomy, education, wealth index, occupation, and urban–rural residence.
