# Supplementary material for: Digital access, transportation, and women’s empowerment in breast cancer screening uptake among Cambodian women: Analysis of the Cambodia demographic and health survey 2021–2022
Source: PLOS Digit Health. 2025 Sep 17;4(9):e0000976. doi: 10.1371/journal.pdig.0000976 (PMC12443266; doi:10.1371/journal.pdig.0000976)
Supplement: Attachment — Submitted filename: reviewer_report.docx [file pdig.0000976.s002.docx]

**Review report for the Digital Access, Transportation, and Women’s Empowerment in Breast Cancer Screening Uptake Among Cambodian Women: Analysis of the Cambodia Demographic and Health Survey 2021-2022**

**Abstract**

**Strengths**

The abstract succinctly summarizes the background, methods, results, and conclusions. The use of statistical results (AOR, 95% CI) improves scientific rigor.

**Suggestions for improvement**

In the abstract, consider clarifying the study design used in the first sentence of the methods to set expectations. Avoid redundancy in the first sentence ("The breast cancer cases are increasing…" could be rephrased for smoother readability, e.g., "Breast cancer incidence is increasing…"). Specify in the abstract whether “breast examination” mainly refers to clinical breast examination or includes mammography; this will reduce ambiguity for readers unfamiliar with the CDHS measurement approach. Streamline the interpretation in the last part of the abstract by focusing on actionable implications rather than repeating results.

**Introduction**

**Strengths**

The introduction provides strong epidemiological context for both global and Cambodian breast cancer burden. Logical flow from general problem to national context, and then to specific gaps in knowledge. Inclusion of references to national strategies strengthens the policy relevance.

**Suggestions for improvement**

The introduction is quite dense; consider shortening sentences to improve readability for an international audience. Clarify how your study builds upon and differs from the previous CDHS analysis cited in [14], be explicit about the novelty. When discussing digital access and transportation, briefly highlight any hypothesized pathways (e.g., improved access to information, reduced travel barriers) to strengthen the conceptual framework. Consider integrating a short paragraph that synthesizes these factors into a clear conceptual model leading to the study objectives.

**Methods**

**Strengths**

Ethical approval and consent procedures are clearly stated. The sampling design is described in detail, including weights, clustering, and stratification. The outcome and independent variables are well-defined with operational categories.

**Suggestions for improvement**

The description of variables and survey design is thorough. Yet, details on how missing data were handled (besides exclusions) could be expanded.

Add clarity on whether all included variables were pre-specified or selected based on bivariate significance.

Explain if ethical approval was needed for secondary data use, even if waived.

Clarify whether “breast examination” responses included both provider-led CBEs and mammography, and whether the survey instrument differentiated them. Specify how missing data were handled beyond the initial exclusion (e.g., for covariates — was complete case analysis used?). Justify why certain potential confounders (e.g., marital status, parity) were excluded, given their known relevance in screening literature. When describing effect modification testing, clarify why age × smartphone ownership was chosen and not tested for other plausible interactions (e.g., media exposure × residence). The Hosmer-Lemeshow test with survey data has limitations — you could note this to preempt reviewer concerns.

**Results**

**Strengths**

Results are clearly structured, moving from descriptive statistics to bivariate associations and multivariable analysis. Tables are detailed and provide both unadjusted and adjusted estimates. Reporting of AORs with CIs and p-values is consistent.

**Suggestions for improvement**

In the descriptive section, consider adding a brief narrative interpretation (e.g., “Most women owned a smartphone, yet only ~11% had been screened…”). Highlight notable unexpected findings in-text (e.g., higher screening uptake in rural vs. urban women in unadjusted analysis) and signal that these are further explored in discussion.

In the multivariable analysis, clarify the rationale for including interaction terms and report model diagnostics (e.g., AUC) more prominently.

Explain why the ROC AUC threshold of 0.653 is described as “moderate” and its implications for the model’s predictive capacity.

**Discussion**

**Strengths**

Interprets findings in the context of Cambodian health policy and global evidence. Thoughtful consideration of both digital and traditional media access as enablers of screening. Addresses the socioeconomic gradient in screening uptake.

Suggestions for improvement:

The discussion is detailed but could benefit from a more concise synthesis of the main findings before moving into interpretations. It should better emphasize how the findings advance current knowledge. Some interpretations (e.g., smartphone vs. non-smartphone similarity) could be expanded by referencing relevant behavioral or technological adoption theories (referencing digital literacy issues more deeply). The rural–urban pattern reversal in unadjusted results warrants brief exploration, even if it becomes non-significant after adjustment — possible role of community outreach or health equity programs? When suggesting mHealth interventions, be explicit about feasibility in the Cambodian context given varying digital literacy levels. Link findings more directly to specific elements of the National Cancer Control Plan and other ongoing initiatives.

**Policy Implications**

**Strengths**

Well-linked to existing national programs like the Health Equity Fund and VHSG networks. Integrates digital strategies with community-based approaches.

Suggestions for improvement:

Consider prioritizing recommendations (short-, medium-, and long-term) for clearer applicability. Specify how proposed strategies might be evaluated for impact.

**Limitations**

**Strengths**

Addresses key methodological constraints (cross-sectional design, recall bias, lack of differentiation in screening type). Acknowledges unmeasured confounders.

**Suggestions for improvement**

Explicitly note that the outcome measure captures “ever” having a breast exam, which may not reflect current or regular screening behavior. Mention the possible influence of COVID-19 service disruptions during the data collection period (Sept 2021–Feb 2022) as a contextual limitation. Acknowledge that travel time as a self-reported measure may be subject to perception bias.

**Conclusion**

**Strengths**

Summarizes the main determinants concisely. Reinforces the policy relevance of the findings.

**Suggestions for improvement**

Add one sentence clearly stating the novel contribution of the study to the literature. Consider ending with a forward-looking perspective (e.g., need for longitudinal or intervention studies to address gaps).

**Overall Assessment**

This is a well-structured and relevant manuscript addressing an important public health issue in Cambodia. The study makes a valuable contribution by linking digital access and empowerment with screening uptake in a LMIC context. With refinements to clarity, contextual interpretation, and explicit positioning of novelty, the manuscript would be strengthened for publication.
