# Supplementary material for: Digital access, transportation, and women’s empowerment in breast cancer screening uptake among Cambodian women: Analysis of the Cambodia demographic and health survey 2021–2022
Source: PLOS Digit Health. 2025 Sep 17;4(9):e0000976. doi: 10.1371/journal.pdig.0000976 (PMC12443266; doi:10.1371/journal.pdig.0000976)
Supplement: Attachment — Submitted filename: Response to Reviewers.docx [file pdig.0000976.s003.docx]

**Authors’ point-by-point responses to the editor and reviewers**

Manuscript ID: PDIG-D-25-00512

Manuscript Title: Digital Access, Transportation, and Women’s Empowerment in Breast Cancer Screening Uptake Among Cambodian Women: Analysis of the Cambodia Demographic and Health Survey 2021-2022

PLOS Digital Health

Dear Dr. Ayatollahi,

Thank you for the opportunity to revise our manuscript and for the thoughtful and constructive comments on our submission. We appreciate the time and effort you have dedicated to improving our paper. We have carefully considered all the feedback and have revised the manuscript to address each point.

A marked-up copy of the manuscript highlights all changes, and an unmarked version is also included for your convenience. We believe the revisions have significantly strengthened the paper and enhanced its clarity and scientific rigor. Below, we provide a point-by-point response to each comment raised.

1. **Journal Requirements**

**Comment:** "Please provide an Author Summary. This should appear in your manuscript between the Abstract (if applicable) and the Introduction, and should be 150–200 words long. The aim should be to make your findings accessible to a wide audience that includes both scientists and non-scientists."

**Author's response**: Thank you for this suggestion. We have added an Author Summary to the revised manuscript, positioned between the Abstract and the Introduction. The summary is approximately 190 words and is written in a clear, non-technical style to be accessible to a wide audience, including non-scientists.

2. **Reviewer Citation**

**Comment:** "If the reviewer comments include a recommendation to cite specific previously published works, please review and evaluate these publications to determine whether they are relevant and should be cited."

**Author's response**: We have reviewed the reviewer comments and found no specific recommendations to cite additional published works.

3. **Reviewer Comments**

**Reviewer #1**

**Comment:** "I suggest clarifying why the study utilized data from the Cambodia Demographic and Health Survey (CDHS) 2021–2022 years exactly. Is it the only available dataset or the last available dataset? Or do you choose it for another reason?"

**Author's response**: Thank you for this important question. We have clarified the rationale for using the CDHS 2021–2022 dataset in the Introduction. The CDHS is the most recent nationally representative survey on health indicators in Cambodia, and this specific dataset contains novel and detailed information on digital access and women's autonomy, which are central to our study's objectives. We have added a sentence to the Introduction to explicitly state that we utilized "***the latest nationally representative and cross-sectional data from the Cambodia Demographic and Health Survey (CDHS) 2021–2022***" to address the identified knowledge gaps.

**Comment:** "In the introduction, you mentioned The National Action Plan for Cervical Cancer 56 Prevention and Control (2019-2023), lines 55-56; I suggest also adding the results or recommendations from this plan."

**Author's response**: We have revised the Introduction to incorporate the recommendations from Cambodia's national health plans. The revised text now notes that the National Action Plan for Cervical Cancer Prevention and Control and the National Cancer Control Plan (2025-2030) "***recommend increasing access to screening services, improving public knowledge through health education, and enhancing communication efforts to achieve national health targets***." This addition strengthens the policy relevance of our manuscript.

**Reviewer #2**

**Comment:** "The introduction sets a solid foundation by outlining the public health importance and Cambodia’s context. However, it would benefit from improved flow and focus. Currently, it is somewhat repetitive and data-heavy… To strengthen the narrative, I recommend a clearer funnel structure: starting broad, then narrowing to national context, screening barriers, potential solutions, research gaps, and finally study aims, ensuring smooth transitions between each section."

**Author's response**: We appreciate this feedback. We have thoroughly revised the Introduction to improve its flow and structure. We have reduced data redundancies and added transitional sentences to create a clearer narrative. The revised Introduction now follows a funnel structure, moving from the global context of breast cancer to the national context in Cambodia, then to specific barriers, potential solutions, and the research gaps our study addresses.

**Comment:** "To improve flow in Introduction, consider adding transitional or framing sentences at the start of thematic paragraphs in the Introduction. For example: “*In addition to informational and economic barriers, physical access remains an important but understudied determinant of screening uptake in Cambodia…*”"

**Author's response**: We have incorporated transitional and framing sentences at the beginning of key paragraphs in the Introduction to improve the flow and help readers understand the rationale for introducing each new factor, such as physical access and women’s autonomy.

**Comment:** "The description of independent variables would benefit from clearer organization… I recommend structuring them as distinct subsections or using clear formatting cues (e.g., bolded subheadings)… Explicitly stating that independent variables are derived from standardized CDHS survey questions… The description of "Digital Access"… currently groups mobile phone ownership, internet use, and media exposure together, which reads like a composite or latent variable. Since these are separate independent variables in the analysis, describing each variable individually and avoiding wording that implies they form a combined index would be helpful."

**Author's response**: We have revised the Methods section to address these points. We have added bolded subheadings for "Digital Access," "Transportation," "Healthcare Access," and "Autonomy in Healthcare Decision-Making" to improve organization. We have explicitly stated that all independent variables were derived from standardized CDHS survey questions. We have also separated the description of the "Digital Access" variables to clarify that they are individual independent variables and not a single composite index.

**Comment:** "If using survey-weighted logistic regression (as indicated by the design-based F-statistics), please add a brief note explaining why F-statistics are reported instead of the usual χ² tests (OR) include “adjusted for survey design” to clarify this for readers."

**Author's response**: We appreciate this important observation. In response, we have added a clarification in the Statistical Analysis section stating that F-statistics are reported instead of the usual Chi-squared tests (Table 2) to properly account for the complex survey design, which incorporates sampling weights, clustering, and stratification. Additionally, we have included the phrase “adjusted for survey design” in the Results section where appropriate to highlight this adjustment for readers.

**Comment:** "Only two interaction terms… were tested, without a stated rationale for their selection… If these were examined but found non-significant, this should be stated for transparency. If not examined, authors should provide a rationale for limiting the analysis to the current two interactions."

**Author's response**: We have provided a clear rationale for selecting the two interaction terms (age × smartphone ownership and media exposure × smartphone ownership). Additionally, we have added a statement in the Methods section clarifying that other plausible interactions—such as wealth × media exposure, education × media exposure, and smartphone ownership × urban/rural residence—were also examined but they were not statistically significant, as these reflect plausible conceptual links between access and empowerment.

**Comment:** "Minor tightening of language to avoid redundancies and clarity on the p-value explanation would make it solid."

**Author's response**: We have reviewed the Discussion section and made minor language adjustments to improve clarity and avoid redundancies, particularly in the explanation of p-values.

**Comment:** "Table 2 has “Ref.” in multiple places, but non-specialist readers might not notice what each variable is compared against. A footnote could help."

**Author's response**: We have added a footnote to Table 2 to explain that "Ref." stands for the reference group, which is the group used for comparison.

**Comment:** "Consider adding bivariant correlation table - a compact table makes it verifiable and easier to compare with adjusted results."

**Author's response**: We acknowledge the reviewer’s suggestion. Table 2 in the manuscript presents both unadjusted (bivariate) and adjusted odds ratios for each independent variable. The unadjusted odds ratios serve as the bivariate correlation, allowing readers to easily compare these results with the adjusted estimates from the multivariable models.

**Reviewer #3**

**Comment:** "In the abstract, consider clarifying the study design used in the first sentence… Avoid redundancy in the first sentence… Specify in the abstract whether “breast examination” mainly refers to clinical breast examination or includes mammography… Streamline the interpretation in the last part of the abstract by focusing on actionable implications rather than repeating results."

**Author's response**: We have revised the Abstract to address all of these suggestions. We have rephrased the first sentence to improve readability and avoid redundancy. We have clarified that “breast examination” refers to both clinical breast examinations (CBEs) and imaging techniques, such as mammograms. We have also streamlined the interpretation at the end to focus on actionable implications.

**Comment:** "The introduction is quite dense; consider shortening sentences to improve readability… Clarify how your study builds upon and differs from the previous CDHS analysis cited in [14]… When discussing digital access and transportation, briefly highlight any hypothesized pathways… Consider integrating a short paragraph that synthesizes these factors into a clear conceptual model leading to the study objectives."

**Author's response**: We have revised the Introduction to shorten sentences and improve readability. We have explicitly stated how our study builds upon previous CDHS analysis by focusing on a more recent dataset and a novel combination of variables (digital access, transportation, and autonomy). We have also added a paragraph that highlights the hypothesized pathways and synthesizes these factors into a conceptual model.

**Comment:** "Details on how missing data were handled (besides exclusions) could be expanded… Add clarity on whether all included variables were pre-specified or selected based on bivariate significance… Explain if ethical approval was needed for secondary data use, even if waived… Clarify whether “breast examination” responses included both provider-led CBEs and mammography… Justify why certain potential confounders… were excluded… Clarify why age × smartphone ownership was chosen and not tested for other plausible interactions."

**Author's response**: We have revised to provide additional clarity on several points:

1. **Missing data:** We specified how missing values were handled; cases with missing outcome data were excluded, and the proportions of missing data for each variable are reported.
2. **Variable selection:** All independent variables were selected based on prior literature and theoretical relevance. Additionally, variables with a bivariate association at p ≤ 0.05 were included in the multivariable models.
3. **Ethical approval:** We clarified that ethical approval for secondary analysis of CDHS 2021–22 data was obtained from the relevant institutional review board, and the publicly available de-identified dataset allowed a waiver of individual consent.
4. **Outcome definition:** The “breast examination” outcome included both provider-conducted clinical breast exams (CBEs) and mammography, as measured using the standard DHS questionnaire
5. **Confounder selection:** Certain potential confounders were social determinants of health related to breast cancer screening. However, we excluded due to the unavailability of data in the secondary dataset.
6. **Interaction term rationale:** The interaction between age and smartphone ownership was selected a priori based on prior evidence indicating that smartphone access may have differential effects across age groups, reflecting both biological and social contexts in Cambodia. Other plausible interactions (e.g., media exposure × smartphone ownership, education × media exposure, rural residence × phone ownership) were also examined but were not statistically significant.

This revision ensures transparency and aligns with standard reporting guidelines.

**Comment:** "In the descriptive section, consider adding a brief narrative interpretation… Highlight notable unexpected findings in-text (e.g., higher screening uptake in rural vs. urban women in unadjusted analysis)… Explain why the ROC AUC threshold of 0.653 is described as “moderate” and its implications for the model’s predictive capacity."

**Author's response**: We have added a brief narrative interpretation to the descriptive section to highlight key findings. We have also highlighted the higher screening uptake in rural women in the unadjusted analysis and noted that this will be further explored in the Discussion. We have included an explanation of the ROC AUC value, stating that the value of 0.653 indicates a moderate predictive capacity for our model.

**Comment:** "The discussion… could benefit from a more concise synthesis of the main findings before moving into interpretations. It should better emphasize how the findings advance current knowledge… The rural–urban pattern reversal in unadjusted results warrants brief exploration… When suggesting mHealth interventions, be explicit about feasibility in the Cambodian context… Link findings more directly to specific elements of the National Cancer Control Plan and other ongoing initiatives."

**Author's response**: We apologize for the earlier typographical error regarding rural women having a higher proportion of breast cancer screening compared to urban women. This has been corrected in the revised manuscript. We have also revised the discussion to begin with a concise synthesis of the main findings. Our revisions explicitly emphasize how our findings advance current knowledge, particularly regarding the influence of digital access, media exposure, and transportation on breast cancer screening uptake. We included a brief discussion of the rural–urban pattern in screening and clarified the feasibility of mHealth interventions in the context of Cambodia’s digital literacy. Additionally, we have linked our findings more directly to specific elements of the National Cancer Control Plan and other ongoing initiatives in Cambodia.

**Comment:** "Consider prioritizing recommendations (short-, medium-, and long-term) for clearer applicability. Specify how proposed strategies might be evaluated for impact."

**Author's response**: We have revised the Policy Implications section to prioritize our recommendations into short- and long-term strategies for clearer applicability. We have also added suggestions on how these strategies might be evaluated for impact.

**Comment:** "Explicitly note that the outcome measure captures “ever” having a breast exam… Mention the possible influence of COVID-19 service disruptions… Acknowledge that travel time as a self-reported measure may be subject to perception bias."

**Author's response**: We have added these specific limitations to the Limitations section of the manuscript. The revised text now explicitly notes that the outcome measure captures "ever" having a breast exam, the possible influence of COVID-19 disruptions, and that the self-reported travel time may be subject to perception bias.

**Comment:** "Add one sentence clearly stating the novel contribution of the study to the literature. Consider ending with a forward-looking perspective (e.g., need for longitudinal or intervention studies to address gaps)."

**Author's response**: We have revised the Conclusion to include a clear statement of the study's novel contribution. We have also added a forward-looking perspective, suggesting the need for future longitudinal or intervention studies to address the identified gaps.

We hope that these revisions adequately address all of the concerns and suggestions raised by the editor and reviewers. We believe the manuscript is now much improved and is suitable for publication in PLOS Digital Health. We are grateful for your time and consideration.

Sincerely,

**Samnang Um and Co-authors**
